# Comparison of treatment results between microvascular decompression and gamma knife radiosurgery in primary trigeminal neuralgia

**DOI:** 10.1097/MD.0000000000039626

**Published:** 2024-09-06

**Authors:** Moon Seok Yang, Chang Hwa Choi, Jae Meen Lee

**Affiliations:** a Department of Neurosurgery, BHS Hanseo Hospital, Busan, Republic of Korea; b Department of Neurosurgery, Busan Bumin Hospital, Busan, Republic of Korea; c Department of Neurosurgery and Biomedical Research Institute, Pusan National University Hospital, Busan, Republic of Korea.

**Keywords:** gamma knife radiosurgery, microvascular decompression, trigeminal neuralgia, Visual Analog Scale

## Abstract

This study aims to analyze and evaluate the comparative clinical outcomes associated with microvascular decompression (MVD) and gamma knife radiosurgery (GKRS), focusing on pain relief, pain recurrence, and complications encountered in the treatment of trigeminal neuralgia (TN). Among 155 surgical procedures performed for TN (90 GKRS, and 65 MVD) between March 1997 and December 2020, the exclusion criteria encompassed prior surgical interventions, the presence of other pathological conditions such as tumors, vascular diseases, and multiple sclerosis, as well as patients who were lost to follow-up. Ultimately, 101 patients received their initial treatment for primary TN (47 GKRS, and 54 MVD) and were followed up for more than 1 year. The MVD procedures utilized the suboccipital retrosigmoid sinus approach, whereas GKRS was conducted with MR imaging guidance, employing a single 4mm isocenter, with median GKRS doses of 80 Gy. We retrospectively analyzed patient characteristics, including sites of divisions, distributions of pain, and clinical outcomes. The assessment of outcomes was performed utilizing the Barrow Neurological Institute Pain Intensity Score and the Visual Analog Scale (VAS), with evaluations taking place preoperatively and after 1, 3, 6 and 12 months. Postoperative VAS scores for patients undergoing either MVD or GKRS demonstrated a significant improvement when compared with their preoperative counterparts. The reduction in postoperative VAS scores within the MVD group was significantly more substantial than that observed in patients who underwent GKRS at the initial postoperative evaluations (*P* = .037). The maintenance of pain relief after MVD proved significantly superior to that following GKRS (*P* < .01). Both MVD and GKRS present as safe and efficacious therapeutic options for individuals diagnosed with primary TN, though MVD displayed superior initial outcomes in terms of pain relief and its maintenance. However, for older patients or those with medical contraindications to invasive procedures, GKRS emerges as a viable and less invasive alternative for initial treatment in cases of primary TN.

## 1. Introduction

Trigeminal neuralgia (TN) is characterized as a debilitating neurological condition, marked by severe, episodic facial pain with a profound impact on quality of life.^[1–4]^ The condition’s etiology typically involves vascular compression of the trigeminal nerve, though additional factors, such as demyelination and genetic predispositions, play a role in a subset of patients (3.1–17%).^[5,6]^ The management of TN, especially when resistant to pharmacological interventions, poses a formidable challenge in clinical practice.

Microvascular decompression (MVD) and gamma knife radiosurgery (GKRS) constitute the principal surgical treatments for TN.^[3,4,7–9]^ MVD, which is more invasive, directly addresses TN’s underlying cause by relieving the trigeminal nerve’s vascular compression, thereby aiming for immediate and long-lasting pain mitigation, often recommended for younger patients in good health.^[10–12]^

Alternatively, GKRS offers a noninvasive approach by employing focused radiation to the trigeminal nerve root.^[7–9]^ This method is especially suitable for elderly individuals or those unsuitable for surgery. Although GKRS’s effects on pain relief manifest over weeks to months, its favorable safety profile and reduced complication risk position it as a critical component of TN’s surgical management spectrum.

This investigation endeavors to compare the clinical outcomes, specifically pain relief, recurrence of pain, and complications associated with MVD and GKRS, in primary TN management.

## 2. Materials and methods

### 2.1. Patients

This retrospective study analyzed 155 surgical interventions for TN conducted between March 1997 and December 2020, comprising 90 GKRS and 65 MVD. Exclusion criteria included prior procedures, the presence of other pathological conditions such as tumors, vascular diseases, and multiple sclerosis, as well as loss to follow-up. Ultimately, 101 individuals undergoing first-time treatment for primary TN (47 GKRS and 54 MVD) with more than 1 year of follow-up were included in this study. Patient demographics, clinical outcomes, and pain localizations were retrospectively analyzed. The Institutional Review Board of Pusan National University Hospital sanctioned this study (H-2201-002-110).

### 2.2. Pain assessment

In this study, postoperative outcomes were assessed using the Visual Analog Scales (VAS) score,^[13]^ even though the Barrow Neurological Institute pain scale is more commonly employed.^[14]^ “Effective pain relief” was categorized as a VAS score of <5, while “Significant pain relief” involved a reduction in the VAS score by 5 or more. Clinical outcomes at follow-ups were further categorized into 3 groups: good (VAS 0–3), fair (VAS 4–6), and poor (VAS 7–10).

### 2.3. Pre- and postoperative evaluations

Patients’ characteristics, including sites, divisions, and distributions of pain as well as clinical outcomes, were assessed retrospectively. Using VAS scores, we evaluated the outcomes preoperatively and postoperatively at intervals of 1, 3, 6, and 12 months, and further, at 2, 3, and 5 years. After GKRS and MVD, trigeminal nerve sensory dysfunction reported by patients was categorized as either bothersome or mild.

### 2.4. Radiosurgical procedures

**Figure 1.**
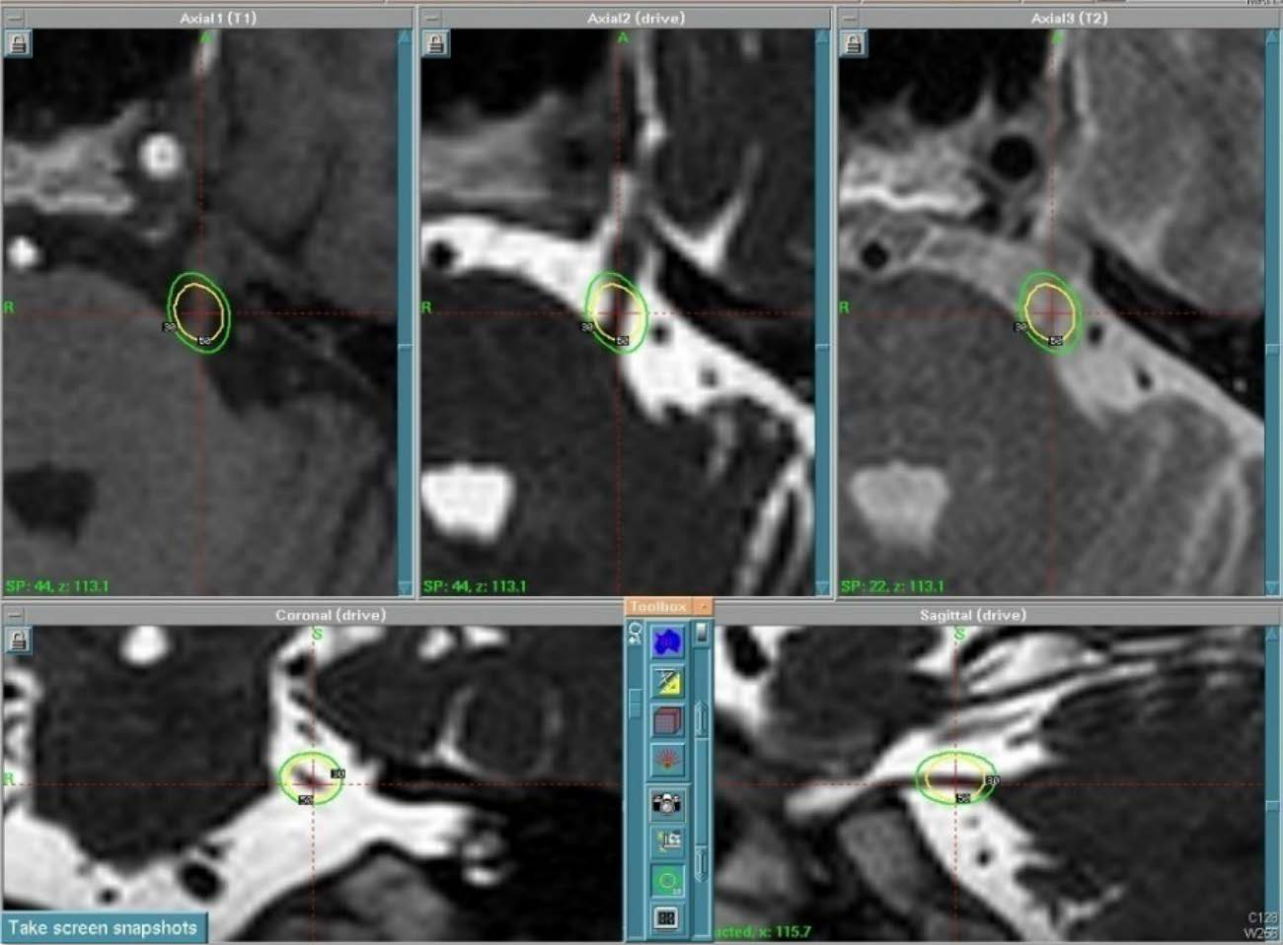
Gamma knife radiosurgery isodose distributions including the root entry zone (REZ) of the trigeminal nerve immediately adjacent to the pons.

All GKRS procedures were performed utilizing the Leksell Gamma Knife (models B and Perfexion; Elekta, Stockholm, Sweden). Dose planning for each patient involved a 0.6 mm axial slice interval with no gap, utilizing CISS. Treatment protocol encompassed a single shot of radiation prescribed between 80 and 85 Gy (average 80.5 Gy) to the maximum dose point using a 4-mm collimator, with or without a shield. The Leksell GammaPlan treatment planning system (Elekta) facilitated the generation of treatment plans. The trigeminal target was the root entry zone (REZ) of the TN close to the pons (Fig. 1).

### 2.5. Surgical procedures

All MVD operations were executed via a lateral retrosigmoid suboccipital approach as previously described.^[15–18]^ Employing an operating microscope, the arachnoid membrane was carefully dissected to expose the REZ, relax the brain, and minimize traction on the trigeminal nerve. A Teflon felt (IMPRA, Inc., A Subsidiary of C. R. Bard, Inc., Norwalk, CA) was then placed between the REZ and the offending blood artery to identify the vessel loop responsible for compression.^[19]^

### 2.6. Statistical analysis

Data analysis was performed utilizing SPSS 25.0 (SPSS Inc., Chicago, IL). Results were presented as percentages and mean ± standard deviation. Statistical comparisons were drawn using Student *t* test, Fisher exact probability test, and Chi-squared tests. Kaplan–Meier survival curves illustrated the duration to achieve pain relief following MVD and GKRS. A *P* value of <.05 (two-tailed) was designated to signify statistical significance.

## 3. Results

### 3.1. Patient characteristics

The mean ages at surgery were 50.65 (range, 36–78 years) and 65.50 years (range, 30–87 years), with mean preoperative symptom durations of 52.93 (range, 2–216 months) and 45.19 months (range, 1–120 months), respectively. The predominant pattern of pain affected both the V2 and V3 distributions of the trigeminal nerve (Table 1). In the MVD group, the mean age was 58.65 years (range, 36–78 years), and in the GKRS group, it was 67.5 years (range, 30–87 years); age did not differ significantly between groups (*P* = .082). The mean symptom duration was 52.93 months (range, 2–216 months) for the MVD cohort and 45.19 months (range, 1–120 months) for the GKRS group. The mean VAS scores were 7.09 (range, 4–10) for the MVD group and 7.72 (range, 6–10) for the GKRS group, showing a significant difference between the groups (*P* = .025). Hypertension and diabetes mellitus did not show significant differences between the groups. Notably, no patients in the MVD group had a history of myocardial infarction, compared to 6.4% in the GKRS group (*P* = .097). Additionally, a history of cancer treatment was noted in 1.9% of the MVD group and 10.6% of the GKRS group (*P* = .094).

**Table 1:** Demographic and clinical characteristics of the study subjects.

	MVD	GKRS	*P* value
Sex (M/F)	20/34	11/36	.293
Side (Rt/Lt)	28/26	31/16	.143
Mean age in years (range)	58.65 (36–78)	67.50 (30–87)	.082
Mean symptom duration in months (range)	52.93 (2–216)	45.19 (1–120)	.450
Mean VAS score (range)	7.09 (4–10)	7.72 (6–10)	.025*
Hypertension (%)	16 (29.6)	12 (25.5)	.646
Diabetes mellitus (%)	6 (11.1)	5 (10.6)	.939
History of myocardial infarction (%)	0	3 (6.4)	.097
History of cancer treatment (%)	1 (1.9)	5 (10.6)	.094
*Pain distribution*			
V1	3	3	
V2	15	11	
V3	11	10	
V1 + V2	4	7	
V2 + V3	18	13	
V1 + V2 + V3	3	3	
Sensory dysfunction (%)	3 (5.6)	3 (10.6)	>.999

GKRS = gamma knife radiosurgery, MVD = microvascular decompression, VAS = Visual Analog Scale.

* Indicates statistical significance.

### 3.2. Pain relief and complications

**Figure 2.**
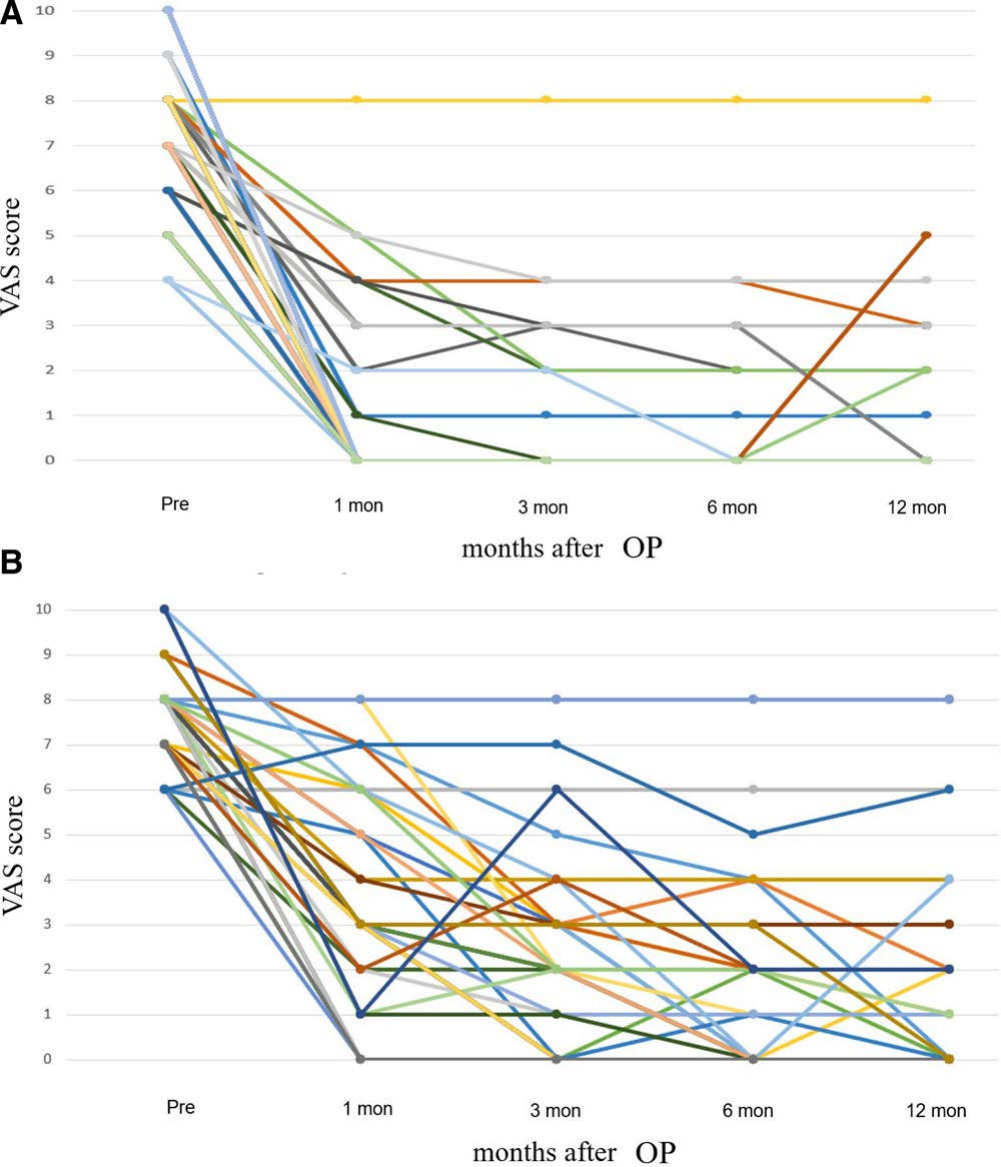
VAS scores at each follow-up session for patients undergoing MVD and GKRS. (A) MVD and (B) GKRS. GKRS = gamma knife radiosurgery, MVD = microvascular decompression.

**Figure 3.**
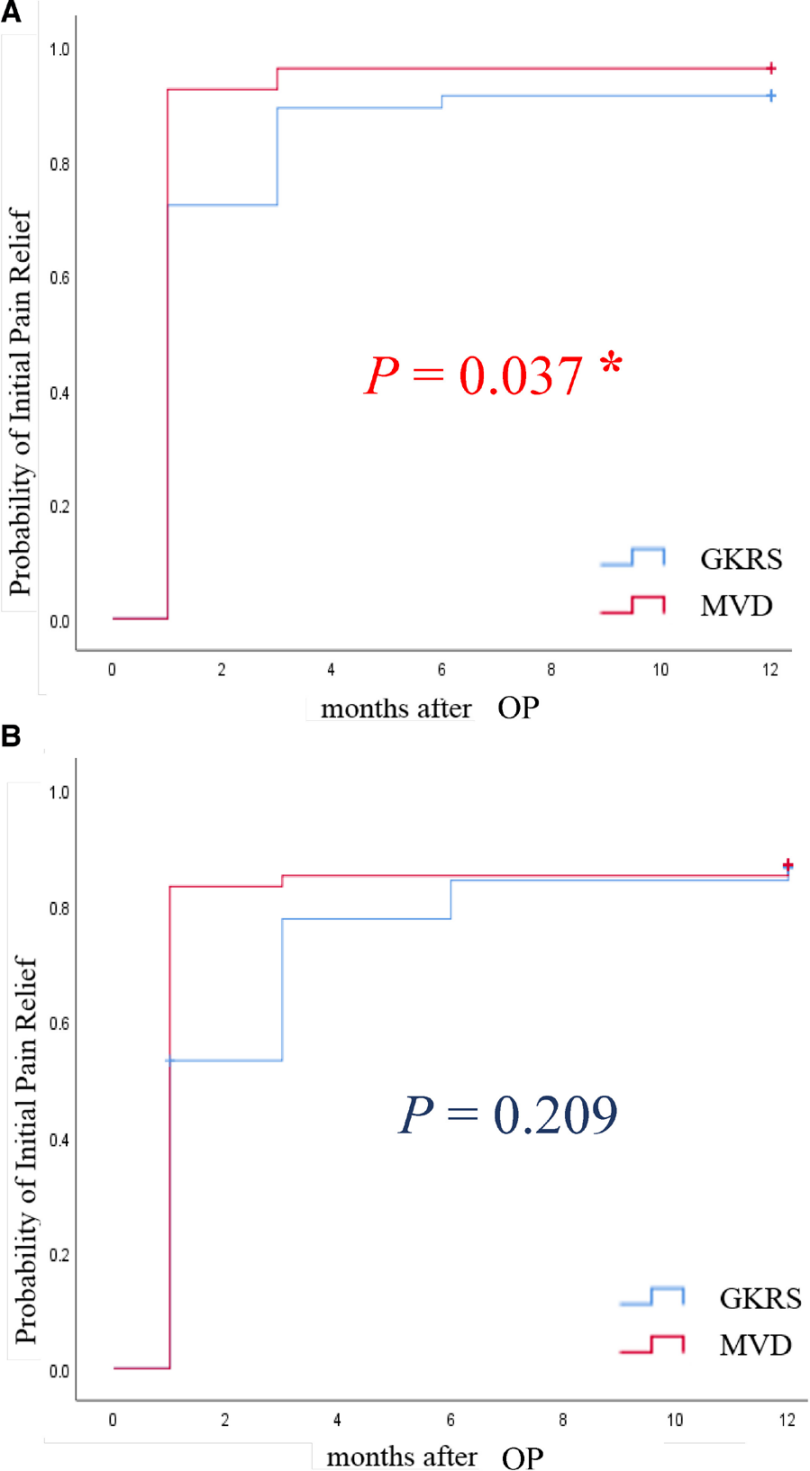
Kaplan–Meier estimates of the probabilities of initial pain relief after MVD and GKRS. (A) Effective pain relief (VAS score < 5), (B) Significant pain relief (a VAS score reduction of ≥5). GKRS = gamma knife radiosurgery, MVD = microvascular decompression, VAS = Visual Analog Scales. * indicates statistical significance.

**Figure 4.**
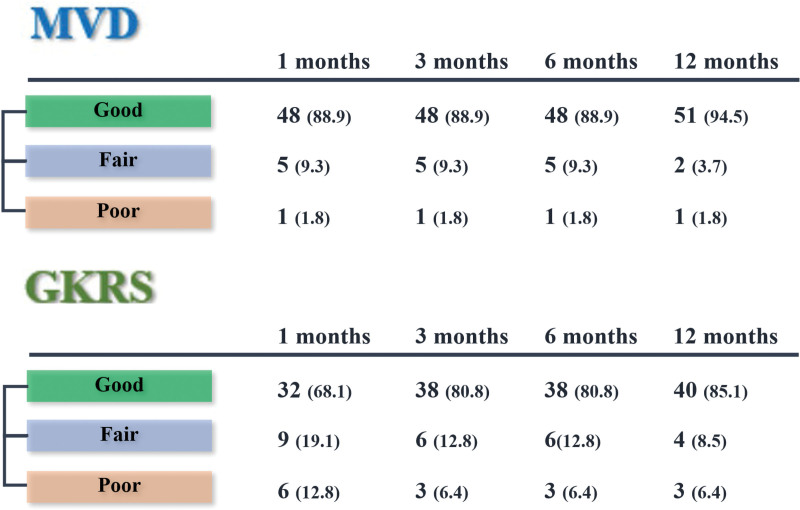
Outcomes of MVD and GKRS over 12 months as measured by Visual Analog Scale Scores (number [%]). MVD = microvascular decompression, GKRS = gamma knife radiosurgery.

**Figure 5.**
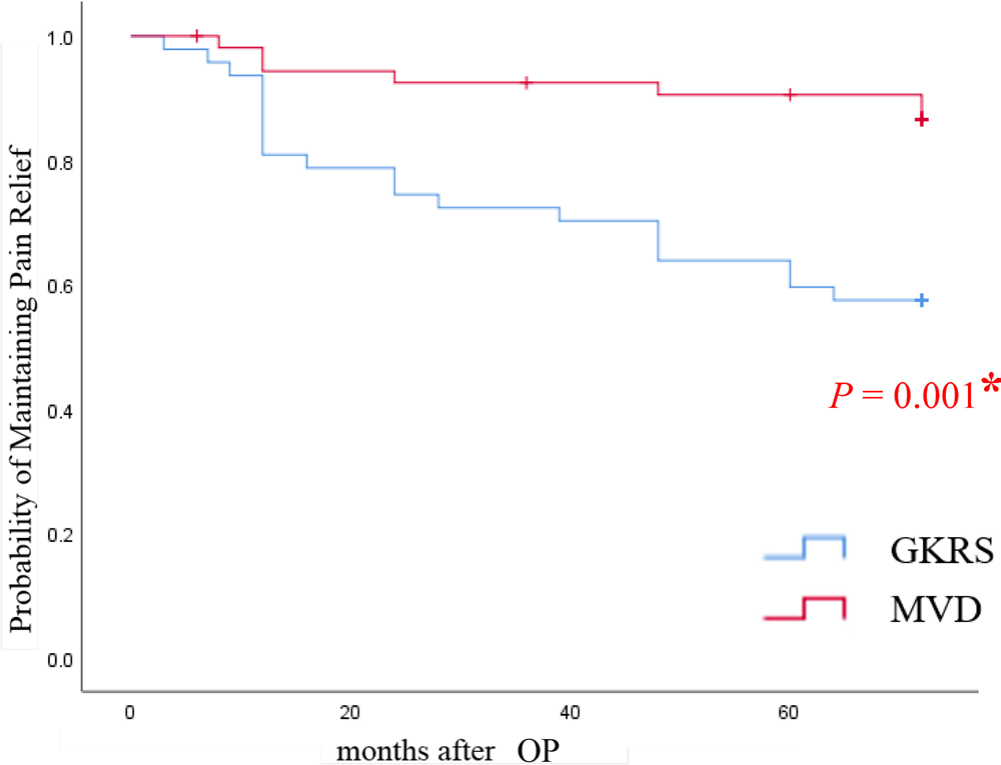
Kaplan–Meier estimates for probabilities of maintaining pain relief following GKRS. GKRS = gamma knife radiosurgery, MVD = microvascular decompression. * indicates statistical significance.

VAS scores at each follow-up for individual patients are presented in Figure 2. Postoperative VAS scores for patients treated with either MVD or GKRS showed significant improvement compared with preoperative scores. Pain relief, as quantified using the Kaplan–Meier method, revealed that “effective pain relief” occurred sooner post-GKRS, whereas “significant pain relief” was more delayed (Fig. 3). Initial postoperative VAS scores for the MVD group were significantly lower than those for the GKRS group (*P* = .037), indicating a faster rate of pain relief with MVD (Fig. 3). The 1-year outcomes for MVD were 94.5% (good), 3.7% (fair), and 1.8% (poor), while for GKRS they were 85.1% (good), 8.5% (fair), and 6.4% (poor), respectively (Fig. 4). Maintaining pain relief following MVD was significantly greater than that following GKRS (*P* < .01) (Fig. 5).

Up to 6 months post-GKRS, no additional sensory disturbances were reported. However, at the 1-year mark, 3 GKRS patients (10.64%) developed facial paresthesia. Following MVD, 3 patients (5.56%) experienced sensory abnormalities. The incidences of postoperative facial numbness did not significantly differ between GKRS and MVD patients (*P* > .999) (Table 1). Additionally, postoperative cerebrospinal fluid leakage occurred in 3 cases, but all patients fully recovered without requiring special treatment.

## 4. Discussion

TN is typically induced by the extrinsic compression of the REZ, often attributable to a normal cerebral vessel.^[1,20]^ This compression results in focal demyelination and deformation of the nerve root.^[20]^ The defining symptoms of TN, described as ’intense, paroxysmal, brief, electric, or stabbing unilateral facial pain following the dermatomal distribution of the 5th cranial nerve’,^[21]^ are paramount for accurate diagnosis. The efficacy of MVD in treating TN largely depends on the re-myelination of nerve fibers following the decompression of the REZ. Similarly, GKRS is reported to be highly effective, offering a safe and relatively complication-free alternative compared to other surgical methods.

The current study assesses the short-term and long-term efficacy of both MVD and GKRS, scrutinizing their safety profiles and impacts on patient quality of life. Such analysis is crucial for informed clinical decision-making, particularly given the significant impact of choosing between MVD and GKRS on patient prognoses. Given TN’s chronic and recurrent nature, comprehending the longevity of pain relief, recurrence rates, and risks associated with each treatment module is fundamental.

### 4.1. Efficacy of MVD and GKRS in TN treatment

This study was designed to assess the efficacy and safety of MVD and GKRS in the treatment of trigeminal neuralgia (TN), particularly among patients with conditions refractory to medical treatment. The outcomes demonstrate that both MVD and GKRS markedly alleviate pain, thereby confirming their utility as effective treatments for TN.

### 4.2. MVD: superior initial and sustained pain relief

Our study substantiates the claims in the current body of literature, showing that MVD delivers unparalleled initial alleviation from TN symptoms when contrasted with GKRS.^[22–24]^ This intervention, by alleviating or excising vessels in proximity to the trigeminal nerve, tackles the underlying presumed cause of the neuralgia directly. The efficacy of MVD is fundamentally ascribed to the resolution of neurovascular conflict, culminating in prompt and lasting mitigation of pain. Our observations align with those of Jannetta et al, who initially posited the neurovascular compression hypothesis and verified MVD’s effectiveness in ameliorating TN.^[25]^ Additionally, our data endorse the notion that MVD not only secures impressive rates of initial analgesia but also preserves these benefits over time, which bears particular relevance for younger patients seeking a durable resolution to their symptoms.

Despite MVD’s efficacy in TN treatment, the absence of neurovascular compression is a recognized limitation, with reports indicating that around 20% of TN patients lack an identifiable offending vessel.^[6,26]^ Moreover, MVD carries potential complications including infection, cerebrospinal fluid leakage, and risks associated with general anesthesia.^[27]^ Minor complications may encompass headache, nausea, dizziness, auditory changes, and facial paresthesia, with headache and dizziness emerging as the predominant issues.^[28,29]^ In this research, post-MVD sensory disturbances of the trigeminal nerve were the sole complications recorded. However, postoperative cerebrospinal fluid leakage also occurred, and this potential complication should be considered when selecting the appropriate treatment option.

### 4.3. GKRS: effective and safe for high-risk populations

GKRS offers a minimally invasive alternative, particularly for patient populations for whom surgical interventions pose substantial risks. It utilizes focused radiation to precisely target the root of the trigeminal nerve, thereby gradually diminishing pain.^[30]^ Nonetheless, in contrast to MVD, GKRS does not provide immediate pain relief, as the radiation’s effects often require weeks to months to mitigate neuralgia. Our research suggests that GKRS is an important initial treatment option, particularly suited for elderly or medically compromised patients who face a greater risk with more invasive surgeries like MVD. The noninvasiveness of GKRS, coupled with its minimal immediate postoperative complications, enhances its safety profile, rendering it a preferable choice for these patient groups.

Our results highlight GKRS as an effective primary treatment option, offering a less risky approach for patients deemed unfit for the more invasive MVD. This is in agreement with the findings of studies, such as that by Kondziolka et al,^[31]^ which documented adequate pain management with minimal complications. GKRS has been demonstrated to be effective in managing TN in scenarios lacking vascular compression. Conversely, patients with TN due to vascular compression of the nerve see less pain relief from GKRS compared to those without such compression.^[32]^

Kondziolka et al^[31]^ suggest that an optimal total radiation dose varies between 70 Gy and 90 Gy. Pain relief rates markedly decrease with doses under 70 Gy, and doses exceeding 90 Gy are associated with increased postoperative complications.^[33–35]^ Therefore, we provided our patients with an average radiation dose of 80 Gy, yielding significantly positive outcomes that were comparable to those obtained with MVD.

### 4.4. Comparative analysis and patient-centric approaches

While MVD and GKRS are both effective, our study emphasizes the significance of choosing between these treatments by conducting a thorough evaluation of patient-specific factors such as age, overall health, pain intensity, and individual risk profiles. This patient-centric approach ensures that treatment decisions are meticulously tailored to meet the patients’ medical requirements and personal circumstances, thereby optimizing clinical outcomes and enriching patient satisfaction. In our study, older patients and those with a history of cancer treatment or myocardial infarction, who were considered to have higher surgical risks, were more often considered for GKRS. Although the VAS scores for GKRS were higher, averaging in the 7-point range, all patients reported significant pain levels. Thus, the degree of pain was not a determining factor in the choice of procedure.

Our comparative analysis concentrates on the effectiveness of MVD and GKRS in managing trigeminal neuralgia, with a particular focus on the sustainability of pain reduction and the initial decrease in pain levels. The data reveal that MVD provides more prolonged pain relief compared to GKRS. Nonetheless, the initial effectiveness of both treatments in reducing pain, defined as a drop of 5 or more points on the VAS, is similar. Importantly, MVD achieves a rapid decrease in VAS scores significantly swifter, offering prompt relief, which is essential for patients suffering from acute pain episodes. Thus, although both treatments are effective initially, MVD facilitates a faster onset of relief, rendering it particularly suitable for promptly managing symptoms in trigeminal neuralgia. Long-term outcomes further indicate that patients undergoing MVD maintain pain relief for more extended periods than those treated with GKRS, a factor that may guide decisions regarding optimal surgical intervention, particularly advantageous for younger patients or those desiring prolonged symptom-free intervals. Another key consideration is the posttreatment quality of life; studies have demonstrated that successful MVD can markedly enhance quality of life due to its enduring pain relief.

Furthermore, our study sheds light on the relative efficacies and safety profiles of MVD and GKRS. For instance, although MVD provides more immediate and potentially more enduring pain relief, its invasive nature and associated risks may render it unsuitable for certain patients. In contrast, GKRS, despite its slower relief provision, poses fewer surgical complication risks and is especially appropriate for older patients or those less capable of undergoing invasive procedures.^[12,14,36,37]^

### 4.5. Limitations

This study is subject to numerous limitations that merit consideration. First and foremost, the variability in follow-up periods among patients could impact the uniformity of outcomes. Additionally, the retrospective design of our study resulted in some incomplete clinical evaluations. Moreover, the intrinsic constraints of a retrospective analysis diminish the trustworthiness of our findings. The absence of random assignment to MVD versus GKRS introduces potential selection bias. Lastly, our exclusive use of the VAS as the pain measurement tool, excluding the Barrow Neurological Institute score due to patients’ reluctance to discontinue medications, may have influenced the reported improvement in pain, potentially muddling the treatment effects. Nonetheless, the influence of these limitations on the primary conclusions of this report is judged to be minimal.

### 4.6. Future directions in research and practice

Further investigations are crucial to deepen our understanding of the long-term outcomes and the comparative effectiveness and safety of GKRS and MVD in treating trigeminal neuralgia, particularly focusing on pain recurrence and management of complications. Prospective studies, ideally randomized controlled trials, are necessary to furnish more conclusive evidence. Progress in imaging and surgical techniques is also expected to augment the accuracy and success of both treatments, which will assist in refining patient selection and developing treatment strategies that foster personalized and secure management of this disorder.

## 5. Conclusion

Both MVD and GKRS offer safe and efficacious treatments for patients with primary TN, with MVD showing superior initial outcomes in pain relief and maintenance of pain relief. However, GKRS remains a viable and considerable option for primary TN patients as an initial treatment, particularly for the elderly or medically frail, due to its less invasive nature. Especially for patients with a history of surgical risks, such as prior cancer treatment or myocardial infarction, GKRS is a safer option and is anticipated to yield comparably effective outcomes.

## Author contributions

**Conceptualization:** Jae Meen Lee.

**Data curation:** Moon Seok Yang, Jae Meen Lee, Chang Hwa Choi.

**Formal analysis:** Jae Meen Lee.

**Methodology:** Jae Meen Lee.

**Writing – original draft:** Moon Seok Yang, Jae Meen Lee, Chang Hwa Choi.
